# Obesity, insulin resistance, and the pathway to type 2 diabetes

**DOI:** 10.1186/1753-6561-6-S3-O1

**Published:** 2012-06-01

**Authors:** Richard N Bergman

**Affiliations:** 1 Cedars-Sinai Medical Center, Los Angeles, USA

## 

The increase in obesity in the U.S. and elsewhere can be explained by a variety of factors, as outlined by David Allison and his colleagues. Not only is the fast food diet a factor, but also are changes in ambient temperature, obesity-causing psychoactive molecules, associative sorting, changes in smoking habits, and reduction in sleep. In particular is the increase in visceral fat, which is associated with insulin resistance. We have shown in experimental animals that omentectomy increases insulin sensitivity implicating visceral fat *per se* as an important factor. We have followed longitudinally changes in organ function and crosstalk in a large animal model, rendered overweight by a high fat, hypercaloric diet. In normal animals, like in normal humans, there is a wide range of adiposity as well as visceral and subcutaneous fat deposition. Feeding fat increases visceral and subcutaneous fat with little change in body weight. Physiologic changes occur with absolutely no change in fasting glucose: most predictive of insulin resistance is relative reduction in metabolic clearance of insulin by liver, and a modest increase in beta cell sensitivity to glucose. The latter changes result in hyperinsulinemia. Change in insulin sensitivity is also predicted by change in liver insulin clearance, implicating metabolic clearance of insulin as the primary factor in development of the insulin resistance syndrome.

